# The association of maternal vaginal bleeding and progesterone supplementation in early pregnancy with offspring outcomes: a prospective cohort study

**DOI:** 10.1186/s12884-022-04711-1

**Published:** 2022-05-05

**Authors:** Chunrong Zhong, Guoping Xiong, Lixia Lin, Qian Li, Xi Chen, Xu Zhang, Yu Zhang, Shangzhi Xu, Xiaoyi Wang, Duan Gao, Meng Wu, Sen Yang, Weizhen Han, Guoqiang Sun, Xuefeng Yang, Liping Hao, Zhichun Jin, Nianhong Yang

**Affiliations:** 1 Department of Nutrition and Food Hygiene, Hubei Key Laboratory of Food Nutrition and Safety, MOE Key Laboratory of Environment and Health, School of Public Health, Tongji Medical College, Huazhong University of Science and Technology, 13 Hangkong Road, Wuhan, 430030 Hubei China; 2 Department of Obstetrics and Gynecology, The Central Hospital of Wuhan, Wuhan, China; 3 Department of Obstetrics, Maternal, Child Health Hospital of Hubei Province, Wuhan, China; 4 Department of Integrated Traditional & Western Medicine, Maternal, Child Health Hospital of Hubei Province, Wuhan, China

**Keywords:** Vaginal bleeding, Progesterone, Preterm, Small-for-gestational-age, Low birth weight, Weight for age z-score

## Abstract

**Background:**

Progesterone is widely used to improve the adverse pregnancy outcomes related to vaginal bleeding during early pregnancy. However, the evidence of its effectiveness is equivocal.

**Methods:**

Six thousand six hundred fifteen mother-infant pairs from Tongji Maternal and Child Health Cohort (TMCHC) were involved in the study. Information on vaginal bleeding, progesterone administration in early pregnancy were obtained at enrolment. Birth outcomes were obtained from the hospital notes. Body weight of the infants at 12 months of age was collected by telephone interview. Multivariable logistic regression was conducted to estimate the effect of vaginal bleeding and progesterone administration in early pregnancy on birth outcomes and weight status of infants at 12 months of age.

**Results:**

21.4% (1418/6615) participants experienced bleeding in early pregnancy, and 47.5% (674/1418) of them were treated with progesterone. There were no significant associations between progesterone supplementation in early pregnancy and offspring outcomes. Compared to women without bleeding or any therapy, women with bleeding and progesterone therapy experienced increased risk of preterm (OR 1.74, 95% CI 1.21–2.52), and delivering a small-for-gestational-age (SGA) (OR 1.46, 95% CI 1.07–1.98) or low birth weight (LBW) (OR 2.10, 95% CI 1.25–3.51) neonate, and offspring of them had an increased risk of weight for age z-score (WAZ) < -1 at 12 months of age (OR 1.79, 95%CI 1.01–3.19).

**Conclusions:**

Offspring of mothers with bleeding and progesterone therapy were more likely to be a premature, SGA or LBW neonate, and had lower weight at 12 months of age. Progesterone supplementation may have no beneficial effect on improving adverse offspring outcomes related to early vaginal bleeding.

**Trial registration:**

TMCHC was registered at clinicaltrials.gov as NCT03099837 on 4 April 2017.

**Supplementary Information:**

The online version contains supplementary material available at 10.1186/s12884-022-04711-1.

## Background

Vaginal bleeding, a symptom of threatened miscarriage in early pregnancy, occurs in about 20% of recognized pregnancies before the 20^th^ week of gestation and nearly half of these events miscarried [1]. Emerging evidence suggested that episodes of vaginal bleeding in early pregnancy was associated with increased risk of adverse antenatal complications [2], such as preterm [3–6], small-for-gestational-age (SGA) [3], and low birth weight (LBW) [3, 6], while previous evidence also showed that light bleeding or bleeding 1 day during early pregnancy was not associated with reduced birth weight [7, 8].

Progesterone is an immunomodulatory molecule produced by the corpus luteum in the onset of early pregnancy [9], and plays an important role in maintaining pregnancy by helping the fertilized egg to implant in the uterus successfully [10]. It is theoretically plausible that progesterone supplementation during early pregnancy may improve adverse antenatal complications. However, thus far the evidence about the effect of progesterone supplementation on birth outcomes was controversial. Previous research stated that progesterone supplementation in early pregnancy might have a beneficial effect on preventing preterm and improving infant health [11–13]. But several recent randomized trials with a large sample size reported that progesterone supplementation during early pregnancy was not associated with a reduced risk of preterm before 34 weeks of gestation [14–17]. They also observed no difference in neonatal outcomes and live birthweight between progesterone supplementation group and placebo group [15, 16]. In addition, little is known about the long-term effect of vaginal bleeding and progesterone supplementation on the offspring’s growth.

The present study aimed to determine the independent and combined effect of early bleeding and progesterone supplementation on birth outcomes and the growth of infants in the first year of life through a prospective cohort study.

## Methods

### Study design and participants

The study was embedded in the ongoing Tongji Maternal and Child Health Cohort (TMCHC, registered at clinicaltrials.gov as NCT03099837), which is a population-based prospective cohort study designed to investigate the short- and long- term effect of exposures in the pregnancy on the health of mother-infant pairs. From January 2013 to May 2016, eligible participants were invited to join the TMCHC study at their first prenatal care visit in three research hospitals of Wuhan, China.

Of 7843 pregnant women joined the TMCHC study at 10–16 weeks of gestation who reported the detail information about vaginal bleeding and medical treatments in early pregnancy, after excluding those with polycystic ovarian syndrome (*n* = 49), test-tube baby (*n* = 38), twin births (*n* = 142), triplets (*n* = 1), miscarriages (*n* = 160), stillbirths (*n* = 3), taking Chinese medicine in early pregnancy (*n* = 216), missing gestational weight gain (*n* = 49) and information at birth (*n* = 570), 6615 participants were included to examine the association of maternal vaginal bleeding and progesterone supplementation in early pregnancy with the risk of adverse birth outcomes. 3181 infants’ weight at 12 months of age were followed up and were eligible to investigate the effect of maternal early vaginal bleeding and progesterone supplementation on the growth of infants in the first year of life.

### Data collection

At the time of enrolment, all participants completed a structured questionnaire including sociodemographic characteristics (maternal age, ethnology, education, income. etc.), obstetrical information (parity, history of spontaneous and induced abortion, gestational age, last menstrual period, etc.), medical characteristics (history of medical issues, family history of disease, etc.), lifestyle (pre-gravid smoking and drinking habits, etc.) and pre-gravid weight. Maternal height and weight were accurately measured at the same time.

The information about vaginal bleeding and progesterone supplementation were retrospectively collected at enrolment. Participants were instructed to report whether vaginal bleeding occurred, and were asked: “Have you ever taken progesterone for therapeutic purposes since pregnancy?”, if “Yes”, the diagnosis of disease or symptom, the start date and duration of progesterone treatment were recorded. Most of the participants took progesterone for treating vaginal bleeding, and the others took progesterone due to low progesterone level in early pregnancy.

Participants were followed up regularly to have their body weights measured and other health information collected by medical examination. Information on birth outcomes (gestational age at birth, neonatal gender, birthweight, birth length, etc.) were obtained immediately after delivery from the hospital notes. Gestational age was calculated by self-reported last menstrual period at enrolment and confirmed by ultrasound examination at 11–14 weeks of gestation. Pre-pregnancy body mass index (BMI, kg/m^2^) was calculated from self-reported pre-gravid weight and height measured at the first antenatal visit. Subjects were categorized as underweight (BMI < 18.5), normal weight (18.5 ≤ BMI < 24.0), overweight or obese (BMI ≥ 24.0) accordingly [18]. The gestational weight gain was calculated as the difference between the last measured pre-natal weight and pre-gravid weight.

### Outcomes

Preterm and very preterm were defined as a birth prior to 37 and 34 weeks of gestation respectively. LBW was defined as birth weight less than 2500 g regardless of gestational age. SGA was defined as a neonatal birthweight < 10th percentile for gestational age, according to a Chinese neonatal reference [19]. Weight for age z-score (WAZ) was calculated according to 2006 WHO child growth standards [20].

### Statistical analysis

Participants were divided into four groups according to the status of vaginal bleeding and treatment in early pregnancy: 1) women without bleeding and any treatment; 2) women without bleeding but taking progesterone; 3) women with bleeding but untreated; 4) women with bleeding and treated with progesterone. The demographics characteristics were compared among four groups. Descriptive statistics were summarized as mean ± SD for continuous variables and frequency (n) and percentages (%) for categorical variables. Differences of characteristics were assessed by one-way analysis of variance (ANOVA) for continuous variables and chi-square test for categorical variables. Multivariable logistic regression was conducted to estimate the independent and combined effect of vaginal bleeding and progesterone supplementation in early pregnancy on offspring outcomes including very preterm, preterm, SGA, LBW, and WAZ < -1 at 12 months of age. In order to investigate whether progesterone therapy in early pregnancy can improve offspring outcomes among women with bleeding, women with bleeding and non-treatment were used as reference group and multivariable logistic regression was performed to calculate the odd ratios (ORs) with 95% Confidence Intervals (95%CIs) for offspring outcomes.

Gestational weight gain, gestational age at delivery, fetal gender, birth weight, birth length, and breast feeding are often strong predictors of offspring’s growth and were therefore considered as potential confounders. However, these variables might lie on the causal pathway from exposure to outcomes. Therefore, we calculated the risk in different models. Model I was adjusted for maternal age, pre-pregnancy BMI, nulliparity, gravidity, history of spontaneous abortion, history of induced abortion, drinking before pregnancy, and gestational age at enrollment. Gestational weight gain and fetal gender were further adjusted in model II to calculate ORs(95%CIs) of very preterm and preterm. Gestational age at delivery, Gestational weight gain, and fetal gender were also adjusted when calculated the ORs (95%CIs) of SGA, and LBW in model II. OR(95%CI) of WAZ < -1 at 12 months of age in model II was also further adjusted for gestational age at delivery, gestational weight gain, fetal gender, birth weight, birth length, and any breastfeeding at 12 months of age. In our study, there were small amounts of missing data in birth length (*n* = 28), and any breastfeeding at 12 months of age (*n* = 45), therefore we used multiple imputation to deal with missing data when assessed the association between exposures and infants’ growth, and then presented the results of pooled analyses.

To eliminate the potential effect of maternal pre-pregnancy BMI, age, parity and preterm on offspring outcomes and reinforce the validity of our results, complementary analyses were conducted by multivariable logistic regression to investigate the association of maternal bleeding and progesterone therapy in early pregnancy with offspring outcomes among women with pre-pregnancy BMI 18.5–23.9 kg/m^2^, age < 35 years, primiparity and term pregnancy (≥ 37 weeks of gestation).

All the statistical analyses were performed using SAS 9.4 software (Statistics Analysis System, USA), and *P* < 0.05 was considered statistically significant.

## Results

Of the 6615 pregnant women, 1418 (21.4%) reported vaginal bleeding and 47.5% (674/1418) of them treated with progesterone in early pregnancy. In those without bleeding in early pregnancy (5197 of the 6615), 7.2% (376/5197) reported taking progesterone due to low progesterone level in early pregnancy. The median (interquartile range) of gestational age at the beginning of vaginal bleeding was 6 (4, 7) weeks and the duration of progesterone supplementation was 15 (7, 30) days. Participants baseline characteristics are shown in Table 1. Most characteristics were similar among groups, except those women with bleeding were more likely to be older and multiparas, with a history of spontaneous and induced abortion, drinking before pregnancy, and women treated with progesterone were more likely to have a higher pre-pregnancy BMI and a history of spontaneous abortion than those untreated.

**Table 1:** Demographics characteristics of the study population according to vaginal bleeding and progesterone treated in early pregnancy(*n* = 6615)

Characteristics	Overall (*n* = 6615)	No bleeding in early pregnancy		Bleeding in early pregnancy		*P*
		Non-treatment (*n* = 4821)	Progesterone treatment(*n* = 376)	Non-treatment (*n* = 744)	Progesterone treatment(*n* = 674)	
Gestational age at enrollment, wk	13.1(1.4)	13.1(1.4)	12.8(1.1)	13.0(1.3)	13.0(1.4)	< 0.001
Ethnology (Han Chinese)	6456(97.6)	4709(97.7)	361(96.0)	723(97.2)	663(98.3)	0.092
Pre-pregnancy BMI, kg/m^2^	20.8(2.7)	20.6(2.6)	21.5(3.1)	20.9(2.7)	21.2(3.1)	< 0.001
Maternal age, y	28.2(3.5)	28.0(3.4)	28.1(3.4)	28.4(3.6)	28.8(3.6)	< 0.001
Education (≥ 16 schooling years)	3606(54.5)	2626(54.5)	227(60.4)	392(52.7)	361(53.6)	0.091
Average personal income (≥ 5000 CNY)	3759(58.1)	2755(58.5)	202(55.2)	421(57.3)	381(57.9)	0.614
Nulliparity (Yes)	5598(84.6)	4090(84.8)	338(89.9)	604(81.2)	566(84.0)	0.002
Gravidity(*n* = 1)	3823(57.8)	2920(60.6)	202(53.7)	368(49.5)	333(49.4)	< 0.001
History of spontaneous abortion (Yes)	653(9.9)	412(8.6)	54(14.4)	84(11.3)	103(15.3)	< 0.001
History of induced abortion (Yes)	1705(25.8)	1156(24.0)	94(25.0)	251(33.7)	204(30.3)	< 0.001
Smoking before pregnancy (Yes)	240(3.6)	179(3.7)	10(2.7)	27(3.6)	24(3.6)	0.773
Drinking before pregnancy (Yes)	116(1.8)	67(1.4)	11(2.9)	22(2.9)	16(2.4)	0.002
Gestational age at delivery, wk	39.1(1.5)	39.2(1.4)	39.1(1.5)	38.8(1.8)	38.9(1.5)	< 0.001
Gestational weight gain, kg	16.1(4.7)	16.1(4.6)	16.1(5.0)	16.1(4.9)	15.9(5.0)	0.748
Fetal gender (Male)	3559(53.8)	2613(54.2)	200(53.2)	390(52.4)	356(52.8)	0.753
Birth weight, g	3333(442)	3342(426)	3347(464)	3297(497)	3307(471)	0.021
Birth length, cm	50.1(1.5)	50.2(1.4)	50.1(1.4)	49.9(1.7)	50.0(1.7)	< 0.001
Any breastfeeding at 12 months of age (Yes) (*n* = 3136)	1090(34.8)	800(34.8)	64(37.0)	123(34.5)	103(33.7)	0.905
WAZ at 12 months of age (*n* = 3181)	0.78(0.11,1.27)	0.78(0.11,1.21)	0.74(0.11,1.21)	0.72(0.15,1.21)	0.70(0.11,1.21)	0.553

*BMI* body mass index, *CNY* Chinese Yuan, 1 CNY ≈ 0.16US$. WAZ: weight for age z-scores

Data are expressed as mean (SD), median (interquartile range) or n (%)

The association of vaginal bleeding and progesterone supplementation in early pregnancy with the risk of offspring outcomes were shown in Table 2. After adjustment for confounding factors, women with bleeding in early pregnancy had significant increased risk of very preterm (OR 3.56; 95%CI 1.86–6.83) and preterm (OR 1.97; 95%CI 1.46–2.65) than those without bleeding, regardless of whether progesterone was administered. In unadjusted model, progesterone supplementation in early pregnancy was associated with increased risk of LBW (OR 1.75; 95%CI 1.23–2.51). After adjustment for confounding factors, the significant association between them was not observed (OR 1.63; 95%CI 0.98 -2.72). Among women with bleeding in early pregnancy, no significant associations were found between progesterone treatment in early pregnancy and offspring outcomes in unadjusted models, and the associations remained no significant after adjustment for confounding factors (Table 3).

**Table 2:** Association between early vaginal bleeding and progesterone treatment and the risk of offspring outcomes (*n* = 6615)

Outcomes	Vaginal bleeding		Progesterone	
	No	Yes	Non-treatment	Treatment
Very preterm (< 34 weeks of gestation)				
N(%)	24(0.5)	23(1.6)	35(0.6)	12(1.1)
Unadjusted model	1	3.55(2.00–6.32)	1	1.83(0.95–3.53)
Adjusted model I^*^	1	3.50(1.83–6.68)	1	0.84(0.20–1.78)
Adjusted model II^a^	1	3.56(1.86–6.83)	1	0.85(0.40–1.79)
Preterm (< 37 weeks of gestation)				
N(%)	171(3.3)	90(6.4)	207(3.7)	54(5.1)
Unadjusted model	1	1.99(1.53–2.59)	1	1.40(1.03–1.91)
Adjusted model I^*^	1	1.95(1.45–2.63)	1	0.96(0.68–1.36)
Adjusted model II^a^	1	1.97(1.46–2.65)	1	0.96(0.68–1.37)
SGA				
N(%)	325(6.3)	105(7.4)	351(6.3)	79(7.5)
Unadjusted model	1	1.20(0.95–1.51)	1	1.21(0.94–1.56)
Adjusted model I^*^	1	1.21(0.93–1.57)	1	1.19(0.89–1.60)
Adjusted model II^b^	1	1.23(0.94–1.60)	1	1.19(0.88–1.59)
LBW				
N(%)	104(2.0)	63(4.4)	126(2.3)	41(3.9)
Unadjusted model	1	2.28(1.66–3.13)	1	1.75(1.23–2.51)
Adjusted model I^*^	1	2.13(1.47–3.08)	1	1.18(0.78–1.79)
Adjusted model II^b^	1	1.24(0.78–1.97)	1	1.63(0.98–2.72)
WAZ < -1 at 12 months of age (*n* = 3181)				
N(%)	70(2.8)	29(4.3)	80(3.0)	19(3.9)
Unadjusted model	1	1.56(1.01–2.37)	1	1.33(0.80–2.22)
Adjusted model I^*^	1	1.53(0.93–2.53)	1	1.08(0.60–1.93)
Adjusted model II^c^	1	1.40(0.83–2.35)	1	1.13(0.62–2.05)

Data was shown as N(%) or OR(95%CI)

*SGA* small for gestational age, *LBW* low birth weight, *WAZ* weight for age z-scores

^*^ Model I adjusted for maternal age, pre-pregnancy BMI, nulliparity, gravidity, history of spontaneous abortion, history of induced abortion, drinking before pregnancy, gestational age at enrollment. Progesterone supplementation was also adjusted when assessed the association between vaginal bleeding and outcomes, and vaginal bleeding was adjusted when assess the association between progesterone supplementation and outcomes

^a^Model II adjusted for covariates in model I plus gestational weight gain, and fetal gender

^b^Model II adjusted for covariates in model I plus gestational weight gain, gestational age at delivery and fetal gender

^c^Model II adjusted for covariates in model I plus gestational weight gain, gestational age at delivery, fetal gender, birth weight, birth length, and any breastfeeding at 12 months

**Table 3:** The association of progesterone use in early pregnancy with the risk of offspring outcomes among women with bleeding in early pregnancy. (*n* = 1418)

Outcomes	Bleeding in early pregnancy	
	Non-treatment	Progesterone treatment
Very preterm (< 34 weeks of gestation)		
N(%)	14(1.9)	9(1.3)
Unadjusted model	1	0.71(0.31–1.64)
Adjusted model I^*^	1	0.63(0.27–1.48)
Adjusted model II^a^	1	0.61(0.26–1.43)
Preterm (< 37 weeks of gestation)		
N(%)	52(7.0)	38(5.6)
Unadjusted model	1	0.80(0.52–1.23)
Adjusted model I^*^	1	0.78(0.51–1.21)
Adjusted model II^a^	1	0.77(0.50–1.19)
SGA		
N(%)	51(6.9)	54(8.0)
Unadjusted model	1	1.18(0.80–1.76)
Adjusted model I^*^	1	1.19(0.80–1.79)
Adjusted model II^b^	1	1.18(0.79–1.77)
LBW		
N(%)	32(4.3)	31(4.6)
Unadjusted model	1	1.07(0.65–1.78)
Adjusted model I^*^	1	1.05(0.63–1.75)
Adjusted model II^b^	1	1.83(0.93–3.60)
WAZ < -1 at 12 months of age (*n* = 675)		
N(%)	13(3.6)	16(5.1)
Unadjusted model	1	1.45(0.69–3.06)
Adjusted model I^*^	1	1.43(0.67–3.06)
Adjusted model II^c^	1	1.60(0.72–3.57)

Data was shown as N(%) or OR(95%CI)

*SGA* small for gestational age, *LBW* low birth weight, *WAZ* weight for age z-scores

^*^Model I adjusted for maternal age, pre-pregnancy BMI, nulliparity, gravidity, history of spontaneous abortion, history of induced abortion, drinking before pregnancy, gestational age at enrollment

^a^Model II adjusted for covariates in model I plus gestational weight gain, and fetal gender

^b^Model II adjusted for covariates in model I plus gestational weight gain, gestational age at delivery and fetal gender

^c^Model II adjusted for covariates in model I plus gestational weight gain, gestational age at delivery, fetal gender, birth weight, birth length, and any breastfeeding at 12 months

Table 4 showed the impact of vaginal bleeding with or without progesterone treatment in early pregnancy on offspring outcomes. After adjustment for confounding factors, compared to women without bleeding and any therapy in early pregnancy, women with bleeding and non-treatment had the highest risk for very preterm (OR 4.25; 95%CI 2.13–8.49) and preterm (OR 2.21; 95%CI 1.59–3.07). The risk remained increased in those with bleeding and treated with progesterone in early pregnancy (OR 2.79; 95%CI 1.26–6.18 for very preterm and OR 1.74; 95%CI 1.21–2.52 for preterm), and they also showed higher risk of delivering a SGA neonate (OR 1.46; 95%CI 1.07–1.98) and a LBW neonate (OR 2.10; 95%CI 1.25–3.51), and offspring of them also had an elevated risk of WAZ < -1 (OR 1.79, 95%CI 1.01–3.19) at 12 months of age. There were no significant associations between progesterone administration and birth outcomes among women without bleeding.

**Table 4:** The combined effect of vaginal bleeding and progesterone administration in early pregnancy on offspring outcomes

Outcomes	Overall	No bleeding in early pregnancy		Bleeding in early pregnancy	
		Non-treatment	Progesterone treatment	Non-treatment	Progesterone treatment
Very preterm (< 34 weeks of gestation)					
N (%)	47(0.7)	21(0.4)	3(0.8)	14(1.9)	9(1.3)
Adjusted model I^*^		1	1.69(0.49–5.78)	4.16(2.09–8.26)	2.73(1.24–6.05)
Adjusted model II^a^		1	1.74(0.51–5.98)	4.25(2.13–8.49)	2.79(1.26–6.18)
Preterm (< 37 weeks of gestation)					
N (%)	261(4.0)	155(3.2)	16(4.3)	52(7.0)	38(5.6)
Adjusted model I^*^		1	1.32(0.78–2.25)	2.18(1.57–3.02)	1.74(1.20–2.51)
Adjusted model II^a^		1	1.35(0.79–2.30)	2.21(1.59–3.07)	1.74(1.21–2.52)
SGA					
N (%)	430(6.5)	300(6.2)	25(6.7)	51(6.9)	54(8.0)
Adjusted model I^*^		1	1.17(0.77–1.80)	1.20(0.88–1.63)	1.44(1.06–1.96)
Adjusted model II^b^		1	1.19(0.77–1.82)	1.23(0.89–1.68)	1.46(1.07–1.98)
LBW					
N (%)	167(2.5)	94(2.0)	10(2.7)	32(4.3)	31(4.6)
Adjusted model I^*^		1	1.44(0.74–2.80)	2.30(1.52–3.45)	2.44(1.60–3.70)
Adjusted model II^b^		1	1.39(0.61–3.16)	1.15(0.65–2.01)	2.10(1.25–3.51)
WAZ < -1 at 12 months of age (*n* = 3181)					
N (%)	99(3.1)	67(2.9)	3(1.7)	13(3.6)	16(5.1)
Adjusted model I^*^		1	0.61(0.19–1.97)	1.27(0.69–2.34)	1.84(1.05–3.24)
Adjusted model II^c^		1	0.61(0.19–2.00)	1.11(0.60–2.13)	1.79(1.01–3.19)

Data was shown as N(%) or OR(95%CI). *SGA* small for gestational age, *LBW* low birth weight, *WAZ* weight for age z-scores

^*^Model I adjusted for maternal age, pre-pregnancy BMI, nulliparity, gravidity, history of spontaneous abortion, history of induced abortion, drinking before pregnancy, gestational age at enrollment

^a^Model II adjusted for covariates in model I plus gestational weight gain, and fetal gender

^b^Model II adjusted for covariates in model I plus gestational weight gain, gestational age at delivery and fetal gender

^c^Model II adjusted for covariates in model I plus gestational weight gain, gestational age at delivery, fetal gender, birth weight, birth length, and any breastfeeding at 12 months

In the sensitivity analyses (Table S1), after excluding the potential effect modifications that could affect fetal growth, the risk estimates remained significant for LBW (OR 2.51; 95%CI 1.05–6.00) and WAZ < -1 at 12 months of age (OR 2.47, 95%CI 1.12–5.45) in women experienced vaginal bleeding and treated with progesterone.

## Discussion

In the current study, women with vaginal bleeding in early pregnancy had an elevated risk of very preterm and preterm regardless administration of progesterone. There were no significant associations between progesterone administration and offspring outcomes respectively among women with bleeding or not. The risk of very preterm and preterm birth remained increased in women experienced bleeding and treated with progesterone. In addition, offspring of those women were more likely to be a SGA and LBW at birth and had lower weight at 12 months of age.

In the current study, vaginal bleeding in early pregnancy was associated with an increased risk of preterm, which is consistent to several previous studies [3–6]. We also observed that progesterone therapy during early pregnancy had no effect on reducing the risk of preterm in women with bleeding, which is in line with several recent studies [14, 16]. A double-blind, randomized trial including 1228 participants found no significant association between vaginal progesterone and the decreased risk of preterm birth [14]. The PRISM trial [16] including 2079 women with progesterone and 2074 with placebo suggested that progesterone therapy administered during early pregnancy did not decrease the risk of preterm before 34 weeks of gestation among women with bleeding in early pregnancy. In accordance with the results of previous studies [15, 16], the present study suggested that there were no significant associations between progesterone supplementation in early pregnancy and offspring outcomes including SGA, LBW, and WAZ < -1 at 12 months of age, meaning that progesterone supplementation might be not good for improving birth outcomes.

A prospective study of 3531 women observed that only heavy bleeding but not spotting or slight bleeding in the first trimester was associated with a decrease in mean birth weight, even though they did not find significantly association with the risk of LBW and SGA [20]. A recent study reported that vaginal bleeding more than 1 day during the first trimester was associated with smaller fetal weight independent of associated complication, but the similar effect was not observed among women with bleeding 1 day [8]. Hence, heavy bleeding in early pregnancy may have an adverse effect on fetal growth. In the present study, we observed that women with bleeding but untreated showed no increase in the risk of delivering SGA and LBW, this might be due to untreated women had experienced mild bleeding in the early pregnancy, and those with bleeding and treated with progesterone might experience heavier bleeding which resulted in the increase of the risk of SGA and LBW. Further following up the infants’ weight status at 12 months of age, offspring of women with bleeding and progesterone supplementation in early pregnancy had an elevated risk of WAZ < -1. Exclusion of women with pre-pregnancy BMI < 18.5 kg/m^2^ or ≥ 24.0 kg/m^2^, age ≥ 35 years, multiparity and preterm, offspring of women with bleeding and progesterone treatment in early pregnancy remained a significant increase in the risk of LBW and WAZ < -1 at 12 months of age. Hence, exposure of heavy vaginal bleeding in early pregnancy may have a negative long-term effect against the infant’s growth.

The study had several strengths. Firstly, the information of vaginal bleeding and progesterone administration in early pregnancy were collected at 10–16 weeks’ gestation, which was soon after the event and may minimize the recall bias. Secondly, we estimated the long-term effect of maternal vaginal bleeding and progesterone supplementation in early pregnancy on offspring’s growth. Nevertheless, some limitations of our study also should be considered. For one thing, we obtained data from a prospective cohort study and participants did not report the daily dose of progesterone administration, so it is limited to assess the association of the dose of progesterone administration with birth outcomes. In addition, we lacked the detail information of vaginal blood loss in early pregnancy, so it was unclear whether women with heavier bleeding were more likely to be given progesterone, and we cannot assess the association of the severity of bleeding with birth outcomes. More randomized controlled trials are needed to evaluate the safety and efficacy of progesterone supplementation.

## Conclusion

In summary, vaginal bleeding in early pregnancy may increase the risk of preterm birth, regardless of progesterone treatment. Offspring of mothers with bleeding and progesterone therapy were more likely to be SGA or LBW neonate and had lower weight at 12 months of age. Progesterone supplementation may have no beneficial effect on improving offspring outcomes.

## Supplementary Information

**Additional file 1: Table S1.** The association of vaginal bleeding and progesterone administration in early pregnancy with offspring outcomes among women with pre-pregnancy BMI 18.5-23.9 kg/m^2^, age < 35 years, primiparity and term pregnancy (≥ 37 weeks of gestation) (*n*=3605).

## Data Availability

The data analyzed during the current study are not publicly available due to restrictions as they contain information that could compromise the privacy of research participants but are available encoded from the corresponding author on reasonable request.

## Abbreviations

BMI: Body mass index
CI: Confidence interval
LBW: Low birth weight
OR: Odds ratio
SGA: Small-for-gestational-age
TMCHC: Tongji Maternal and Child Health Cohort
WAZ: Weight for age z-scores
