# Supplementary material for: The association of maternal vaginal bleeding and progesterone supplementation in early pregnancy with offspring outcomes: a prospective cohort study
Source: BMC Pregnancy Childbirth. 2022 May 5;22:390. doi: 10.1186/s12884-022-04711-1 (PMC9074309; doi:10.1186/s12884-022-04711-1)
Supplement: Supplementary file 1 — Additional file 1: Table S1. The association of vaginal bleeding and progesterone administration in early pregnancy with offspring outcomes among women with pre-pregnancy BMI 18.5-23.9 kg/m2, age < 35 years, primiparity and term pregnancy (≥ 37 weeks of gestation) (n=3605). [file 12884_2022_4711_MOESM1_ESM.docx]

Table S1. The association of vaginal bleeding and progesterone administration in early pregnancy with offspring outcomes among women with pre-pregnancy BMI 18.5-23.9 kg/m^2^, age < 35 years, primiparity and term pregnancy (≥ 37 weeks of gestation) (n=3605)

| Outcomes |  | Overall |  | No bleeding in early pregnancy | |  | Bleeding in early pregnancy | |
| --- | --- | --- | --- | --- | --- | --- | --- | --- |
|  |  |  |  | Non-treatment | Progesterone treatment |  | Non-treatment | Progesterone treatment |
| SGA |  |  |  |  |  |  |  |  |
| N (%) |  | 226(6.3) |  | 155(5.8) | 17(7.6) |  | 28(7.3) | 26(7.7) |
| Adjusted model Ⅰ^*^ |  |  |  | 1 | 1.42(0.84-2.38) |  | 1.33(0.88-2.02) | 1.43(0.92-2.20) |
| Adjusted model Ⅱ^a^ |  |  |  | 1 | 1.45(0.86-2.46) |  | 1.36(0.89-2.07) | 1.43(0.93-2.22) |
| LBW |  |  |  |  |  |  |  |  |
| N (%) |  | 37(1.0) |  | 22(0.8) | 1(0.5) |  | 7(1.8) | 7(2.1) |
| Adjusted model Ⅰ^*^ |  |  |  | 1 | 0.58(0.08-4.34) |  | 2.43(1.03-5.75) | 2.72(1.15-6.46) |
| Adjusted model Ⅱ^a^ |  |  |  | 1 | 0.59(0.08-4.48) |  | 2.21(0.93-5.29) | 2.51(1.05-6.00) |
| WAZ<-1 at 12 months of age (n=1769) |  |  |  |  |  |  |  |  |
| N (%) |  | 50(2.8) |  | 33(2.5) | 2(2.0) |  | 6(3.2) | 9(5.6) |
| Adjusted model Ⅰ^*^ |  |  |  | 1 | 0.79(0.19-3.35) |  | 1.29(0.53-3.12) | 2.37(1.11-5.06) |
| Adjusted model Ⅱ^b^ |  |  |  | 1 | 0.74(0.17-3.24) |  | 1.27(0.51-3.19) | 2.47(1.12-5.45) |

Data was shown as N(%) or OR(95%CI).

SGA: small for gestational age, LBW: low birth weight, WAZ: weight for age z-scores.

^*^ Model Ⅰ adjusted for gravidity, history of spontaneous abortion, history of induced abortion, drinking before pregnancy, gestational age at enrollment.

^a^ Model Ⅱ adjusted for covariates in model Ⅰ plus gestational weight gain, gestational age at delivery and fetal gender.

^b^ Model Ⅱ adjusted for covariates in model Ⅰ plus gestational weight gain, gestational age at delivery, fetal gender, birth weight, birth length, and any breastfeeding at 12 months.
